# In memoriam Fritz Krafft (10. Juli 1935 – 9. November 2025)

**DOI:** 10.1002/bewi.70024

**Published:** 2026-07-13

**Authors:** Mitchell G. Ash, Cornelius Borck, Bettina Wahrig

**Affiliations:** ^1^ Institut für Geschichte Universität Wien; ^2^ Institut für Medizingeschichte und Wissenschaftsforschung Universität zu Lübeck; ^3^ Institut für Pharmazie‐ und Wissenschaftsgeschichte Technische Universität Braunschweig

## Abstract

Am 9. November 2025 ist Fritz Krafft, der Gründer und langjährige Herausgeber der *Berichte zur Wissenschaftsgeschichte*, im Alter von 90 Jahren verstorben. Von 1988 bis zu seiner Emeritierung 2000 leitete er das Marburger Institut für Geschichte der Pharmazie und der Naturwissenschaften. Bereits 1978, damals war er Professor für Wissenschaftsgeschichte an der Universität Mainz und Präsident der Gesellschaft für Wissenschaftsgeschichte (GWG), initiierte er diese Zeitschrift – und prägte sie dann über drei Dezennien. Deshalb soll er hier als Herausgeber dieses Organs, als Wissenschaftshistoriker sowie als Akteur in der institutionalisierten Wissenschaftsgeschichte gewürdigt werden. Seine Verdienste um die Pharmaziegeschichte und das Marburger Institut sind von seiner Nachfolgerin Tanja Pommerening schon an anderem Ort gewürdigt worden.

## 1 Fritz Krafft als Wissenschaftshistoriker

Das wissenschaftshistorische Profil von Fritz Krafft zeichnet sich durch eine enorme chronologische und thematische Bandbreite aus.^1^ Im Hinblick auf seine langjährige Herausgebertätigkeit scheint dabei auch sein philologischer Start prägend gewirkt zu haben: Der Hamburger Beamtensohn hatte nach humanistischem Abitur 1955 ein Studium der Klassischen Philologie, Philosophie und Germanistik aufgenommen und 1962 mit der Dissertation „Vergleichende Untersuchungen zu Homer und Hesiod“^2^ als Dr. phil. abgeschlossen. Vielleicht waren es die Vorlesungen bei Bruno Snell und Carl Friedrich von Weizsäcker an der Hamburger Universität, die ihn schon früh auf die Wissenschaftsgeschichte aufmerksam machten, wenigstens entschied sich der Studienstiftler gegen die Klassische Philologie und für eine Assistentenstelle bei Bernhard Sticker am damals gerade neugegründeten Institut für Geschichte der Naturwissenschaften der Universität Hamburg. Das führte mitsamt ergänzenden Semestern Physikstudium zur Habilitation an der Naturwissenschaftlichen Fakultät der Hamburger Universität 1968 mit der Arbeit „Dynamische und statistische Betrachtungsweisen in der antiken Mechanik“^3^, die bereits den Bogen zur wissenschaftlichen Revolution in der Frühen Neuzeit schlug.

1970, also im Erscheinungsjahr der Habilitationsschrift, wurde er mit 35 Jahren auf eine Professur für Geschichte der Naturwissenschaften an der Universität Mainz berufen. Zur selben Zeit begann er in zahlreichen nationalen und internationalen Gremien aktiv zu werden, im Deutschen Kepler‐ und im Kopernikus‐Komitee, als Mitglied der Senatskommission für Humanismusforschung der DFG, als korrespondierendes Mitglied der Académie Internationale d'Histoire des Sciences ab 1971 und im Nationalkomitee der International Union of the History and Philosophy of Science ab 1977. Ebenfalls schon in die Jahre in Mainz fiel seine Zuwahl in die Leopoldina 1984.

In diesen zwei Jahrzehnten zwischen der Habilitation in Hamburg und der Berufung nach Marburg erweiterte Fritz Krafft den historischen Horizont seiner Arbeiten von der Alten Geschichte bis zum Humanismus mit Arbeiten zu Kopernikus, Kepler und Otto von Guericke. Gleichzeitig stieß er an der Mainzer Universität auf Fritz Strassmann und wandte sich daraufhin der Physik und der Chemie im 20. Jahrhundert zu. Seine Biographie des berühmten Entdeckers der Kernspaltung war gleichermaßen wissenschaftshistorisch anspruchsvoll und für breitere Kreise ansprechend.^4^ Zugleich war er dabei einer der Ersten, der die wichtige Rolle von Lise Meitner in der Zusammenarbeit von Otto Hahn und Fritz Strassmann herausstellte.

Methodologisch gesehen verfuhr Fritz Krafft damals wie später konsequent entlang der traditionell etablierten Verbindung von Begriffsgeschichte und Biographie, wie dies beispielsweise seine Habilitationsschrift und das Buch zur antiken Wissenschaft^5^, der Aufsatz über Phosphor von 1969^6^ oder das von ihm 1986 und 1999 herausgegebene Lexikon *Große Naturwissenschaftler* zeigen^7^. Dass er sich auch für Historiographie und Metatheorie interessierte, unterstreichen sein Aufsatz „Der Weg von den Physiken zur Physik an den deutschen Universitäten“^8^ im ersten Heft der *Berichte zur Wissenschaftsgeschichte*, seine Beschäftigung mit der Stellung des Menschen im Kosmos^9^ oder sein späterer Aufsatz „Naturwissenschaftsgeschichte und historische Naturwissenschaften“^10^. Zugleich war er ein aufmerksamer Beobachter der internationalen Debatten. Als der theoretische Physiker Joachim Petzold ihn zum Wintersemester 1981 für ein wissenschaftshistorisches Blockseminar nach Marburg einlud (wo er auch die Pharmaziegeschichte kennenlernte), startete Krafft seine Vorlesung mit einer Einheit über „Wissenschaftstheorie und Wissenschaftsgeschichte“ anhand der damals virulenten Diskussionen über Thomas Kuhn, Jürgen Habermas und Paul Feyerabend und schloss daran mit seinem Konzept des „historischen Erfahrungsraums“ an.^11^

Krafft verwies in seiner damaligen Einführung zwar nicht auf Reinhart Koselleck, aber auch er zielte mit dieser Metapher auf eine Gesamtheit von Geschichte, Kultur und Wissenschaft. Erst aus dieser heraus ließen sich wissenschaftliche Entwicklungen adäquat rekonstruieren. Er hat diesen Ansatz (soweit wir das überblicken) nirgends systematisch anhand einer Fallstudie ausgearbeitet, aber im Rückblick erscheint sie doch als zentrales Motiv seines professionellen Wirkens, vor allem auch als Herausgeber der *Berichte zur Wissenschaftsgeschichte*.

## 2 Fritz Krafft, die Gesellschaft für Wissenschaftsgeschichte (GWG) und die *Berichte zur Wissenschaftsgeschichte*

Als die GWG entstand, hatte Krafft gerade die Assistentenstelle bei Sticker in Hamburg angenommen. Der Gründung war bekanntlich 1964 ein Streit in der Deutschen Gesellschaft für Geschichte der Medizin, Naturwissenschaft und Technik (DGGMNT) vorausgegangen, den die Gruppe um Karl Eduard Rothschuh, Fridolf Kudlien und Heinrich Schipperges zum Anlass nahm, mit ihrer Initiative zugleich die Wissenschaftsgeschichte zu professionalisieren.^12^ Bei ihnen sollte es anstelle von Gesellschaftstagungen mit Referaten von Praktikern und festlichem Rahmenprogramm wissenschaftliche Symposien geben, bei denen Fachvorträge von Spezialisten im Zentrum standen. Programmatisch wählte man für die Gründungstagung 1965 in Münster „Zur Problematik und Methodik der Geistesgeschichte im Rahmen der Wissenschaftsgeschichte“ als Thema und zielte damit auf eine integrierte Ideengeschichte von Wissenschaft. Allerdings wurden die Beiträge der Tagungen nur vereinzelt publiziert,^13^ denn die neue Gesellschaft unterhielt ein Mitteilungsblatt, aber keine eigene Zeitschrift.

Das sollte sich ändern, als Krafft 1977 ihr Präsident wurde. Ziel der von ihm als Organ der GWG gegründeten Zeitschrift *Berichte zur Wissenschaftsgeschichte* war es, die verschiedenen Initiativen in Richtung einer Standortbestimmung der Geschichte der Wissenschaften zu fördern und zu koordinieren. Die Publikation der Symposien der Gesellschaft bildete den Kern des neuen Zeitschriftenprojekts, das mit den Beiträgen des von Krafft in Mainz 1977 organisierten XV. Symposiums „Das Entstehen neuer Wissenschaften in der Neuzeit“ startete. Daneben gab es feste Rubriken wie „Forschungsberichte und Spezial‐Bibliographien“ oder „Dokumentation und Information“ sowie Rezensionen.

In seiner Personalunion von Herausgeber und Präsident hob Krafft in einem kurzen Editorial hervor, dass mit Wissenschaft dabei „nicht, wie heute leider üblich geworden ist, […] *science*, also (exakte) Naturwissenschaften, sondern die Vielfalt der Disziplinen, die vom gegenwärtigen Inhalt des deutschen Begriffs ‚Wissenschaft‘ umfaßt werden“, gemeint sei.^14^ Damit wollte er hoch hinaus und adressierte explizit auch die „,scientific community‘ im internationalen Ausland“:

Eine so verstandene Wissenschaftsgeschichte bemüht sich, die vielfältigen und vielschichtigen Gründe und Voraussetzungen für die jeweiligen Denk‐ und Erkenntnisweisen und ihre Ergebnisse und Folgen aus dem gesamten geistigen und sozialen Geschehen heraus aufzuzeigen, und vermag damit in gewissem Sinne auch die Zukunftsdimension der Gegenwart aufzuschlüsseln.^15^

Das ließ international aufhorchen. Frederick Gregory lobte in *Isis* die Gründung der Zeitschrift als Schritt in Richtung Internationalisierung und Timothy Lenoir vermutete die deutsche Wissenschaftsgeschichte damit „on the verge of a major breakthrough“^16^. Dazu ist es vielleicht nochmals zwanzig Jahre später durch die Gründung des Berliner Max‐Planck‐Instituts gekommen, aber die Wahrnehmung war präzise: 1978 wäre es revolutionär gewesen, Wissenschaftsgeschichte nicht aus den Einzeldisziplinen und ihren Theorien zu rekonstruieren, sondern im kulturellen, ökonomischen und soziokulturellen Kontext – also als Wissenschaft im historischen Erfahrungsraum. Aus dem breiten Spektrum der unter dem Motto der Interdisziplinarität und Internationalität stehenden Symposien der GWG, die regelmäßig in dem jeweiligen Jahrgang der *Berichte* veröffentlicht wurden, sei das 1983 stattfindende XXI. Symposium in Wolfenbüttel hervorgehoben. Unter dem Titel „Vor fünfzig Jahren – Emigration und Immigration von Wissenschaft“ nahm die Gesellschaft die 50. Wiederkehr des „Gesetzes zur Wiederherstellung des Berufsbeamtentums“ zum Anlass, an die Ausgrenzung und Vertreibung von Wissenschaftler:innen aus NS‐Deutschland zu erinnern, und verband diese Erinnerung an Verbrechen und Unrecht mit der Frage nach ihren Folgen für Prozesse und Effekte internationaler wissenschaftlicher Netzwerkbildung.^17^

Wie die GWG wollte auch Fritz Krafft mit seiner Zeitschrift professionell und modern sein, aber zugleich unter dem Dach der Ordinarienuniversität bleiben. In diesem Sinne hat er für die Organisation der *Berichte* das Modell eines Editorial Boards abgelehnt. Immer dort holte er auswärtigen Rat ein, wo er dies für notwendig hielt, um dann über die Annahme oder Ablehnung eines Beitrags selbst zu entscheiden. Ansonsten vertraute er auf seine philologische Kompetenz, denn sein Modell verband mit der Herausgeberschaft eine Gesamtverantwortung für die Zeitschrift bis zum Seitenumbruch und bis zum letzten Komma.

Im Sommer 1989, also kurz bevor die DDR und die alte BRD zu Geschichte wurden, erreichte seine Karriere ihren internationalen Höhepunkt, als er gemeinsam mit seinem Kollegen Christoph Scriba im Auftrag der International Union for History and Philosophy of Science den 18. Internationalen Kongress für Wissenschaftsgeschichte zum Tagungsthema „Science and Political Order/Wissenschaft und Staat“ in Hamburg und München ausrichtete.^18^ Schon 1940 war Deutschland bereits einmal als Tagungsort vorgesehen, was aber wegen des vom NS‐Staat angezettelten Weltkriegs nicht hatte realisiert werden können. Im Vorwort zum Kongress verwiesen die beiden Organisatoren auf diese Vorgeschichte und sie erinnerten vor allem daran, dass der Nationalsozialismus viele Wissenschaftler:innen aus Deutschland in die Emigration gezwungen hatte.^19^ Tatsächlich war dieser Kongress eine der ersten internationalen Plattformen für die Diskussion von Wissenschaft im Nationalsozialismus, über die Rolle der I.G. Farben im Krieg sowie staatliche Eugenik. Auch andere, bis dahin wenig beachtete Themen wie Frauen in der Wissenschaft sowie außereuropäische und postkoloniale Wissenschaftsgeschichte wurden verhandelt.

Dass das Thema „Wissenschaft und Politik“ in dieser vielschichtigen Weise ins Zentrum gestellt wurde, kann heute als Signal dafür gesehen werden, dass damals eine neue Generation in der Wissenschaftsgeschichte antrat, die vieles weitertrieb, was die GWG und Fritz Krafft mit den *Berichten zur Wissenschaftsgeschichte* vorbereitet hatten. Wenige Wochen nach dem Kongress startete für Krafft das erste Semester an seinem neuen Wirkungsort Marburg, wo er sich dann vorrangig für die Integration der Pharmaziegeschichte in die Wissenschaftsgeschichte engagierte. Die Gesellschaft für Wissenschaftsgeschichte ist inzwischen gemeinsam mit der DGGMNT in der Gesellschaft für Geschichte der Wissenschaften, der Medizin und der Technik (GWMT) aufgegangen. Fritz Krafft stand diesem Zusammengehen der beiden Fachgesellschaften skeptisch gegenüber, wohl auch deswegen, weil er die GWG als Refugium spezialisierter Symposien bewahren wollte. Der von ihm gegründeten Zeitschrift bescherte der Zusammenschluss jedoch auch neue Impulse, was nicht zuletzt durch den Titelzusatz *History of Science and Humanities* zum Ausdruck kam. Über diesen Weg der *Berichte zur Wissenschaftsgeschichte*, der hoffentlich fortgesetzt werden kann, hat er sich sicher noch gefreut.

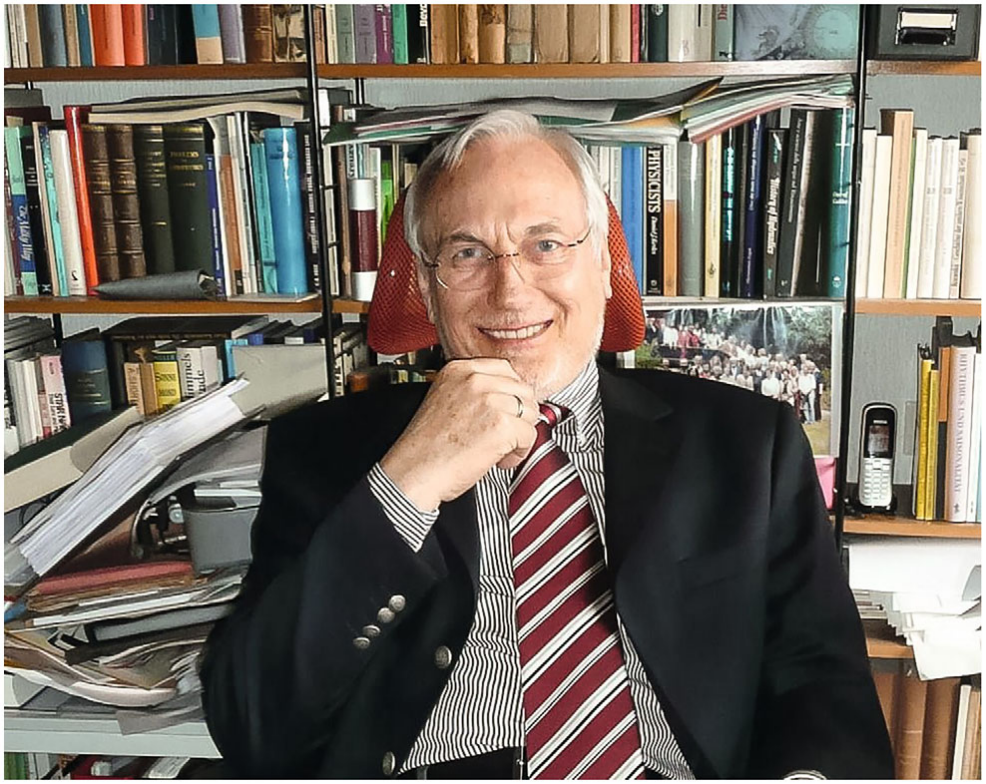

Fritz Krafft (1935–2025).

## Footnotes

1
Pommerening 2025. Die Dokumentation des Festaktes anlässlich seiner Emeritierung enthält ein Schriftenverzeichnis seiner Publikationen von 1958 bis 2002: Schmiederer 2002, S. 89–140.

2
Krafft 1963.

3
Krafft 1970.

4
Krafft 1981.

5
Krafft 1971.

6
Krafft 1969a.

7
Krafft 1986, 1999.

8
Krafft 1978b.

9
Krafft 1982a.

10
Krafft 2000.

11
Krafft 1982b.

12 Anke Jobmann berichtet, dass Rothschuh, Kudlien und Schipperges ihrer Einladung zur Gründungsversammlung an den Kollegen Sticker ausdrücklich hinzufügten, Krafft bitte nicht einzubeziehen, möglicherweise wegen seiner aktiven Rolle bei der DGGMNT (siehe Jobmann 1998, S. 86). Fritz Krafft gehörte zum Driburger Kreis von dessen ersten Anfängen 1962 an (Krafft 1969b), und er wurde von der DGGMNT bei der Würzburger Tagung 1964, die zur Sezession führte, zum Beisitzer in den Vorstand gewählt (siehe *Nachrichtenblatt der DGGMNT* 1964, Titelblattinnenseite).

13 Alwin Diemer z. B. hat das 6. Symposion der Gesellschaft für Wissenschaftsgeschichte 1968 in Fulda zum Thema „Der Wissenschaftsbegriff: Historische und systematische Untersuchungen“ (Diemer 1970) sowie das Symposion „Die Struktur wissenschaftlicher Revolutionen und die Geschichte der Wissenschaften“ 1975 in Münster anlässlich ihres zehnjährigen Bestehens (Diemer 1977) publiziert.

14
Krafft 1978a, S. 1–2.

15 Ebd., S. 3.

16 Zit. nach Wahrig und Ash 2007, S. 7.

17 Siehe Kaiser 1983; Krafft 1984.

18
Krafft und Scriba 1993.

19
Krafft und Scriba 1989, S. 1–2.
